# Obituary

**Published:** 1910-06-15

**Authors:** 


### OBITUARY.

---

Dr. J. Y. Crawford. Died, April 5, 1910, at his home corner Belmont and Acklen aves., Nashville, Tenn., James Young Crawford, M.D., D.D.S., from an attack of angina pectoris.

Dr. Crawford had been subject to angina pectoris for several months. Though physically weak and a constant sufferer, he had continued to give attention to his professional duties until two days before his death, when his condition grew more and more critical, notwithstanding his receiving the best medical attention, and on the morning of the 5th at an early hour he lost consciousness, and all hope of his ultimate recovery was abandoned.

James Young Crawford was a native of Tennessee, his father being a prominent Methodist preacher of that state in ante-bellum days. He was born June 2, 1847. The larger portion of his childhood was spent in West Tennessee, but he had been a resident of Nashville for the past thirty years.

Though only fourteen years of age at the outbreak of the war, he entered the Confederate service, serving in the commissary department in Florida for the four years of that bloody period. At the close of the war he went to Bowling Green, Ky., where he studied dentistry under the preceptorship of one of the leading dentists of that city, later taking his degree at the University of Tennessee with the class of 1878. Following his graduation he located in McKenzie, where he practiced for several years. He then removed to Nashville, where the remainder of his life was spent. He soon took rank as a leader in his profession, and for years he stood without a superior as a dental surgeon in the South.

In the death of Dr. Crawford the dental world has received a vey hard blow, not only account of his ability, but probably more so in that he imbued all who came in contact with him with the extraordinary enthusiasm which he felt for his work. He was an eloquent speaker, and at any meeting he attended Dr. Crawford was sure to be called on to make an address. His friends in the North always spoke of him as the "eloquent Southerner." He was a self-made man, not in the sense that this expression is generally used, for he was a man of gentle birth, but what he accomplished was done through his own efforts. It was owing to ceaseless devotion to his work that he rose to the high place which he occupied at the time of his death. He was a man of influence in his profession, in the social world, and in political life.

Dr. Crawford was the last surviving member of the committee of five prominent dentists who organized the International Dental Congress during the World's Fair at Chicago. He was always a prominent figure in his profession, taking an active interest in its various organizations, and was several times a delegate to the American Dental Association.

He was a good citizen. Possessed of a genial disposition, he had won a large circle of friends not only in the profession which he so long adorned, but among the people generally, who admired him for his charming personality and for the deep interest he ever manifested in the city's welfare. He was a member of the Methodist Church, holding church membership in McKendree at the time of his death. When residing in the West End he was for many years one of the officials of the West End Methodist Church. He was ever ready to contribute liberally of his means to the promotion of the church's work.

In 1885 Dr. Crawford married Miss Louise Haggard, daughter of the late Dr. W. D. Haggard. He is survived by his wife and three children.—May *Cosmos.*

